# Enhanced Photocatalytic Performance of Luminescent g-C_3_N_4_ Photocatalyst in Darkroom

**DOI:** 10.1186/s11671-016-1303-2

**Published:** 2016-02-16

**Authors:** Huihui Li, Shu Yin, Tsugio Sato, Yuhua Wang

**Affiliations:** Key Laboratory for Magnetism Magnetic Materials of the Ministry of Education, Lanzhou University, 222 South Tianshui Road, Lanzhou, 730000 People’s Republic of China; Institute of Multidisciplinary Research for Advanced Materials, Tohoku University, 2-1-1 Katahira, Aoba-ku, Sendai, 980-8577 Japan

**Keywords:** Dye degradation, Water clear, g-C_3_N_4_, Long afterglow assistance, In darkroom

## Abstract

Graphitic-C_3_N_4_(g-C_3_N_4_), a low-cost visible-light-driven photocatalyst, was used for the photocatalytic oxidation of aqueous methylene blue (MB) in the dark with Sr_4_Al_14_O_25_:(Eu,Dy) assistance. The Sr_4_Al_14_O_25_:(Eu,Dy)/g-C_3_N_4_ photocatalysts were fabricated through the ultrasonic dispersion method. The commercial Sr_4_Al_14_O_25_:(Eu,Dy) phosphor was used as a long afterglow supplier for exciting g-C_3_N_4_ in the dark. The results demonstrated that the metal-free g-C_3_N_4_ photocatalyst could use the eye-visible long afterglow to photocatalytically decompose MB dyes in the dark. This work may expand the appealing application of g-C_3_N_4_ for the environmental cleanup.

## Background

The organic dye is one of the most significant identified pollutants in waste waters because of its high toxicity and possible accumulation in the environment [1]. At the same time, the dye, generally as a water-soluble organic colorant, is gradually increased owing to the tremendous increase of industrialization and requirements of human beings for color [2]. The presence of dyes in textile wastewater is an environmental problem due to their high visibility, resistance, and toxic impact [3]. In addition, dyes have direct and indirect toxic effects on humans as they are associated with cancer, jaundice, tumors, skin irritation, allergies, heart defects, and mutations [4], and [5]. Among various treatments, photocatalytic oxidation is found to be one of the most effective ways to degrade dyes in a wastewater and gets rid of their deep color [6].

The photocatalytic performance of semiconductor-based (such as TiO_2_, ZnO, and SrTiO_3_) photocatalysis has received considerable attention for the attractive strategy of conversing solar energy into the formation of hydrogen by the treatment of organics in waste waters [7–9]. However, the lack of visible-light utilization ability and/or the low quantum yield greatly limited their performance and large-scale application. Therefore, designing and optimizing the efficient visible-light responsive photocatalysts have attracted worldwide attention [10, 11]. Ag_3_PO_4_, a breakthrough on visible-light-driven photocatalyst [12], had created the wave of research interest and participation widely [13]. Nevertheless, it was also found that Ag_3_PO_4_ exhibited the low photochemical stability [14, 15], photo-corrosion, [16, 17] and “self-corrosion” characteristic [18], which prevent its use in environment and energy regions. Besides Ag_3_PO_4_, several other visible-light-driven photocatalysts, such as Bi_2_Fe_4_O_9_ [19], Bi_3_NbO_7_ [20], and Bi_2_WO_6_ [21], have also been investigated. To date, however, most of the reported photocatalysts with high photocatalytic ability for water treatment under visible-light irradiation are metal compound-based semiconductors. Considering that metals are relatively expensive materials because of the limited resource, alternative photocatalysts based on precious metal-free materials have been actively pursued [22].

As a metal-free photocatalyst, polymeric graphite-like carbon nitride (g-C_3_N_4_) with a band gap of 2.70 eV has attracted much attention in H_2_ production and contaminants degradation [23–25]. Nevertheless, as other single-phase semiconductors with the narrow band gaps, the high recombination rate of photo-generated electrons and holes of g-C_3_N_4_ restricts its further application in the field of photocatalysis [26]. Up to date, numerous of g-C_3_N_4_-based composites, such as TaON/g-C_3_N_4_ [27], Ag_3_VO_4_/g-C_3_N_4_ [28], and S-TiO_2_/g-C_3_N_4_ [29], have been reported. The hybrid photocatalysts presented much higher activity than the pure one, mainly due to the coupling effect between the g-C_3_N_4_ and semiconductors [30].

On the other hand, the long-lasting phosphorescence, which persists the luminescence for a long time after the removal of the excitation light source, is an interesting phenomenon [31]. Our previous works have revealed that the long afterglow phosphor-assisted Ag_3_PO_4_ photocatalyst can efficiently decompose aqueous organic pollutants even after turning off the irradiation light [16]. However, under lamp irradiation, Ag_3_PO_4_ shows the low chemical stability and photo-corrosion, while this phenomenon does not occur in the g-C_3_N_4_ photocatalytic system. Herein, the present work reports for the first time that the Sr_4_Al_14_O_25_:(Eu,Dy)/g-C_3_N_4_ exhibits good performance on wastewater cleaning both under lamp irradiation and turning off light. This work not only provides a new promising strategy for the full time wastewater purification but also broadens the application of long afterglow phosphors in photocatalysis and deepens the understanding on the combination mechanism of g-C_3_N_4_-based hybrids.

## Methods

All chemicals were purchased from Aladdin (Shanghai, China) except for Sr_4_Al_14_O_25_:(Eu,Dy) powders from Lumin (Dalian, China) and used as received without further purification. The Sr_4_Al_14_O_25_:(Eu,Dy) long afterglow phosphor was used as a phosphorescence assistance because of its stability in aqueous solution for a long time as shown in our previous work [16]. G-C_3_N_4_ was prepared by the thermal polycondensation of melamine [26]. Typically, 5.0 g of melamine in a covered alumina crucible was put into a muffle furnace and heated to 550 °C for 2 h with a heating rate of 10 °C/min. The resulted yellow product was collected and ground into powders. In order to combine Sr_4_Al_14_O_25_:(Eu,Dy) with g-C_3_N_4_, 0.1 g of g-C_3_N_4_ powders were, firstly, dispersed into 10 mL of ammonia solution (25 wt%) via stirring at room temperature for 5 h [26]. The final product was collected by centrifugation and dried at 60 °C under vacuum overnight. Before combination, Sr_4_Al_14_O_25_:(Eu,Dy) and g-C_3_N_4_ were firstly modified by ammonia solution treatment, respectively [26]. Then, Sr_4_Al_14_O_25_:(Eu,Dy)/g-C_3_N_4_ composites were achieved by a simple method [26, 32]. In a typical procedure, 0.12 g of g-C_3_N_4_ powders and 0.08 g of Sr_4_Al_14_O_25_:(Eu,Dy) powders were separately added into 50 mL of methanol and sonicated for 30 min. Then, these two solutions were mixed and continuously stirred in a covered beaker at room temperature for 24 h. After volatilizing the methanol, Sr_4_Al_14_O_25_:(Eu,Dy)/g-C_3_N_4_ powders were obtained after volatilizing methanol and drying. According to this method, the different mass ratios of hybrid photocatalysts were prepared. The obtained composites are named as *x* % composite sheet (CS) (*x* = 20, 40, 60, 80), where *x* refers to the g-C_3_N_4_ weight percent in the composite. For comparison, pure g-C_3_N_4_ and Sr_4_Al_14_O_25_:(Eu,Dy) were similarly treated by methanol and dried, respectively.

The X-ray diffraction (XRD) patterns of the catalysts were measured from 10° to 80° of 2*θ* using a Bruker AXS D2 Phaser X-ray diffractometer and graphite-monochromic CuKα radiation. The catalyst morphology was observed by using an FEI Tecnai G2 F30 transmission electron microscope (TEM) with a Gatan imaging filter (GIF) system. The diffuse reflectance spectra (DRS) were determined using powder samples (PE Lambda 950), and BaSO_4_ was used as a reference. The vibration spectra were characterized by Fourier transform infrared spectroscopy (FT-IR) (NEXUS 670, Nicolet). X-ray photoelectron spectroscopy (XPS) measurement was done using a Kratos AXIS Ultra DLD XPS system with a monochromatic AlKα source and a charge neutralizer; all the binding energies were referenced to the C1s peak at 284.6 eV of the surface adventitious carbon. The photoluminescence (PL) spectra were obtained on a FLS-920T fluorescence spectrophotometer (excitation wavelength 325 nm). The decay curve was then measured with a PR 305 afterglow phosphorescence instrument at 15 °C. The surface charge in aqueous solution was measured using a zeta-potential analyzer (Malvern Zetasizer Nano-ZS 90).

Methylene blue (MB) was taken as a target pollutant to evaluate the photocatalytic activities of the Sr_4_Al_14_O_25_:(Eu,Dy)/g-C_3_N_4_ composites. Sr_4_Al_14_O_25_:(Eu,Dy), a kind of long afterglow phosphor, emits an eye-visible blue and green luminescence with the peak wavelength at *λ* = 490 nm at room temperature. To compare with the long afterglow-assisted photocatalytic activity, a weak-intensity lamp with visible light was used as a light source. Therefore, the visible lamp light-induced photocatalytic reaction experiments were conducted using a 350-W Xe lamp (Au-Light, CEL-LAX 350) equipped with a 420-nm cutoff filter as the visible-light source (ca. 0.11 mW/cm^2^). Typically, a 100 mL of MB aqueous solution at a concentration of 5 mg/L was mixed with 0.08 g of sample in a 500-mL beaker for reaction. Prior to light irradiation, the suspensions were continuously stirred in the dark for 60 min to reach the adsorption/desorption equilibrium. Then, the light was turned on, and 5 mL of the suspension was withdrawn every 20 min, centrifuged, and filtered to remove the solid particles. The filtrates were analyzed by recording variations of the maximum absorption peak (664 nm for MB). The decoloration efficiency was recorded as *C*/*C*_0_, where *C* is the MB concentration after adsorption or photocatalysis and *C*_0_ is the initial concentration.

To investigate the effect of long afterglow assistance on photocatalytic activity, the catalytic reaction was proceeded in the dark for 10 h after the photoexcitation of the sample to generate the long afterglow. Typically, 0.01 g of the sample was first irradiated by an 8-W black lamp for 30 min and then well dispersed in 25 mL MB dye solution (2.5 mg/L) for ca. 10 h in the dark. Then, the sample powders were removed from the solution to determine the concentration of MB dye remained in the solution. After that, the sample was irradiated by the black lamp for 30 min again and put into the MB dye solution in the dark. These procedures were repeated 15 times, i.e., the total reaction time was 150 h. In order to avoid the influence of other light, the photocatalytic reaction proceeded in the darkness with an opaque material hood. The MB dye solution with dispersed photocatalyst sample was put into a 50-mL centrifuge tube with black tapes. The experimental procedure and evaluation of persistent photocatalytic degradation reaction are similar to those in the previous work [16].

## Results and Discussion

**Fig. 1:**
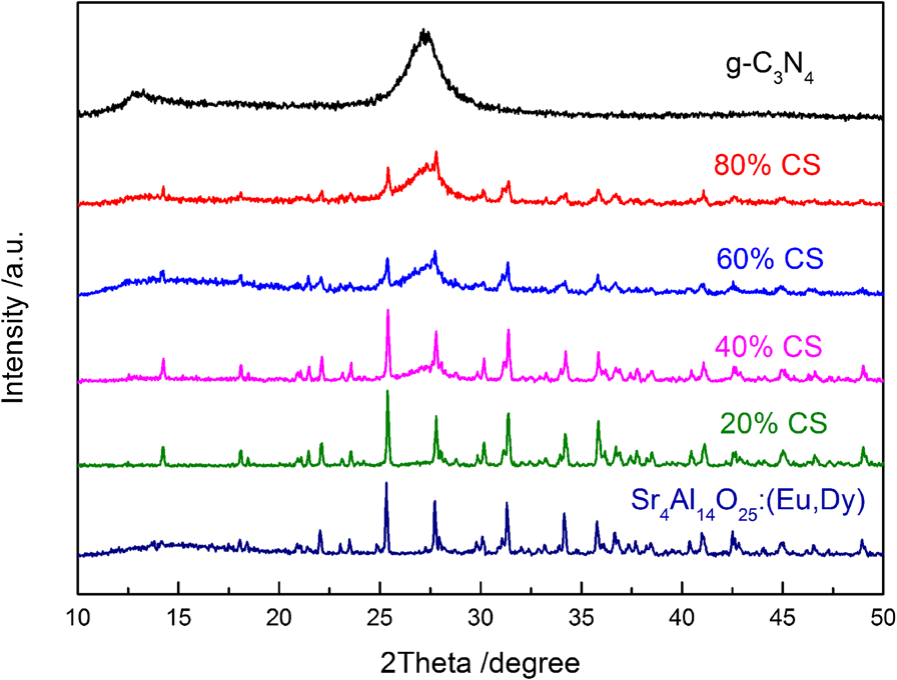
XRD patterns of g-C_3_N_4_, Sr_4_Al_14_O_25_:(Eu,Dy) and various Sr_4_Al_14_O_25_:(Eu,Dy)/g-C_3_N_4_ composites

The XRD patterns of g-C_3_N_4_, Sr_4_Al_14_O_25_:(Eu,Dy) and various Sr_4_Al_14_O_25_:(Eu,Dy)/g-C_3_N_4_ composites are shown in Fig. 1. The peak at 2*θ* = 27.4° for pure g-C_3_N_4_ can be indexed as the (002) diffraction plane of g-C_3_N_4_ (JCPDS no. 87-1526). No obvious changes appear in g-C_3_N_4_ after treating by ammonia solution and then methanol, suggesting that the g-C_3_N_4_ is stable and is not degraded easily during the chemical treatment. The XRD patterns of the Sr_4_Al_14_O_25_:(Eu,Dy)/g-C_3_N_4_ composites with different g-C_3_N_4_ contents are also shown in Fig. 1. Both Sr_4_Al_14_O_25_:(Eu,Dy) and g-C_3_N_4_ are detected. There is no clear crystalline g-C_3_N_4_ in the hybrid samples with low g-C_3_N_4_ content, whereas the peaks of g-C_3_N_4_ in hybrid sample becomes more and more obvious with higher g-C_3_N_4_ content (>40 %), confirming the coexistence of g-C_3_N_4_ and Sr_4_Al_14_O_25_:(Eu,Dy). This result is similar to the previous reports on ZnO·g-C_3_N_4_ composites [32], i.e., the g-C_3_N_4_ particles with low crystallinity were dispersed uniformly on the ZnO surface when the g-C_3_N_4_ content was low, but crystalline g-C_3_N_4_ particles appeared when its content exceeded a threshold value (40 %). It is also seen that with the increase of the g-C_3_N_4_ content, the g-C_3_N_4_ XRD peaks increased and the Sr_4_Al_14_O_25_:(Eu,Dy) peaks decreased.

**Fig. 2:**
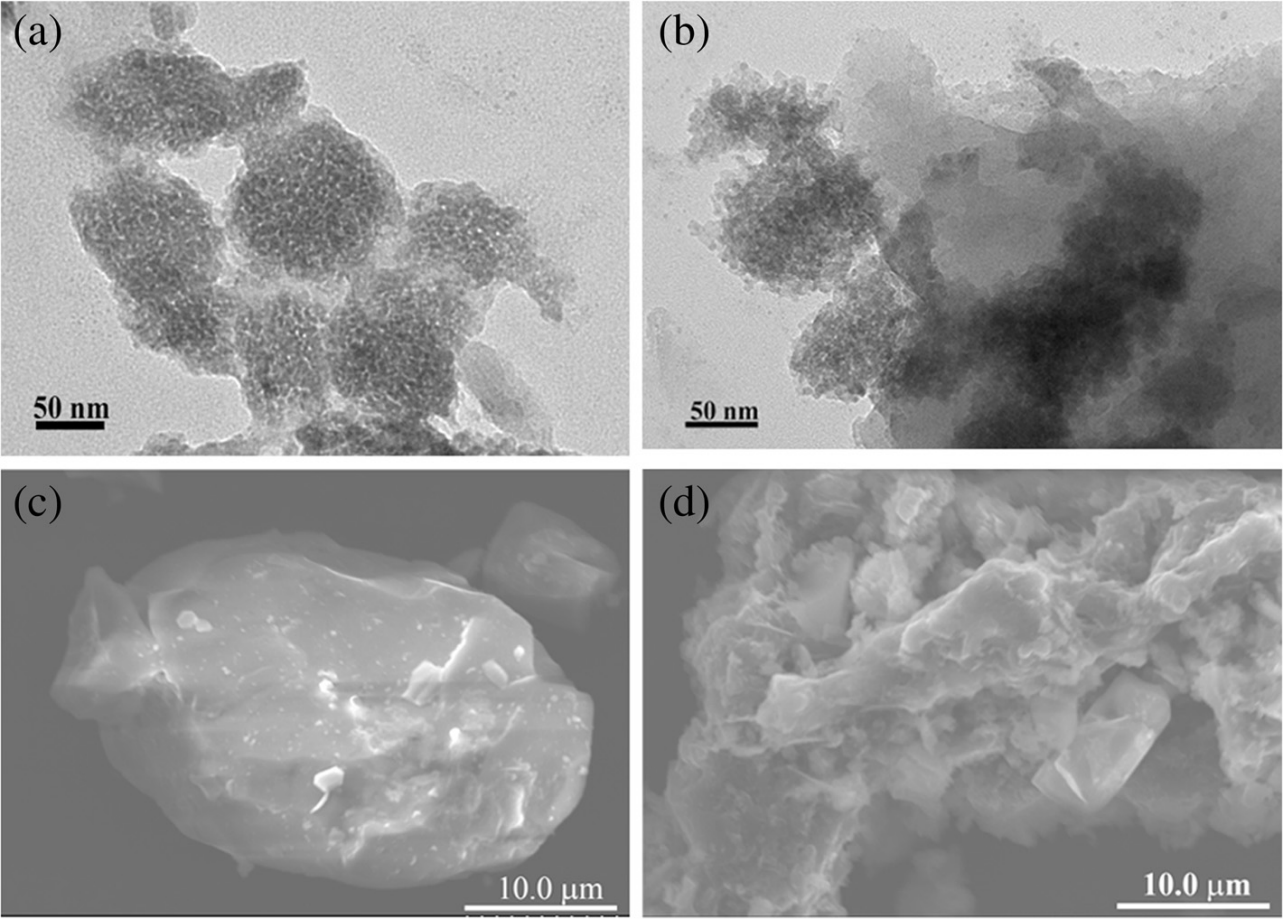
**a** TEM image of g-C_3_N_4_, **b** TEM and **d** SEM images of 60 % CS, and **c** SEM image of Sr_4_Al_14_O_25_:(Eu,Dy)

The morphology and microstructure of as-prepared samples were further investigated by TEM and SEM images. As shown in Fig. 2a, the g-C_3_N_4_ treated by ammonia solution has a nano-sized structure. After coupling with Sr_4_Al_14_O_25_:(Eu,Dy), the g-C_3_N_4_ nano-particles are attached to the surface of the huge irregular shape of Sr_4_Al_14_O_25_:(Eu,Dy) particles (Fig. 2b). The particles with dark color can be ascribed to g-C_3_N_4_, whereas the gray area can be assigned Sr_4_Al_14_O_25_:(Eu,Dy). In addition, it is clear that compared with the bare surface of Sr_4_Al_14_O_25_:(Eu,Dy) particle, the 60 % CS sample shows the g-C_3_N_4_ coated on the Sr_4_Al_14_O_25_:(Eu,Dy) firmly, indicating the formation of a well-structured composite, as shown in Fig. 2c, d.

**Fig. 3:**
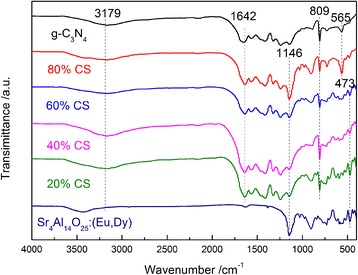
FT-IR spectra of g-C_3_N_4_, Sr_4_Al_14_O_25_:(Eu,Dy), and various Sr_4_Al_14_O_25_:(Eu,Dy)/g-C_3_N_4_ composites

To further observe the effect of the hybrid combination in g-C_3_N_4_-based composites, FT-IR analysis was carried out. As shown in Fig. 3, the g-C_3_N_4_-based samples show the peaks at 1642 and 809 cm^−1^ attributing to the C-N stretching vibration modes and the s-triazine ring system, respectively, and those at 1241, 1319, 1409, and 1569 cm^−1^ originating from the aromatic C-N stretching [26, 28, 32]. The main peaks of pure Sr_4_Al_14_O_25_:(Eu,Dy) also appear in the Sr_4_Al_14_O_25_:(Eu,Dy)/g-C_3_N_4_ composites. In addition, the peaks at 3179 cm^−1^ of g-C_3_N_4_-based samples and 3432 cm^−1^ of Sr_4_Al_14_O_25_:(Eu,Dy) show that there are hydroxyl groups on the surfaces of both g-C_3_N_4_ and Sr_4_Al_14_O_25_:(Eu,Dy). The grafting of hydroxyl groups on the surface of two precursors may be formed during the treatment by the ammonia solution.

**Fig. 4:**
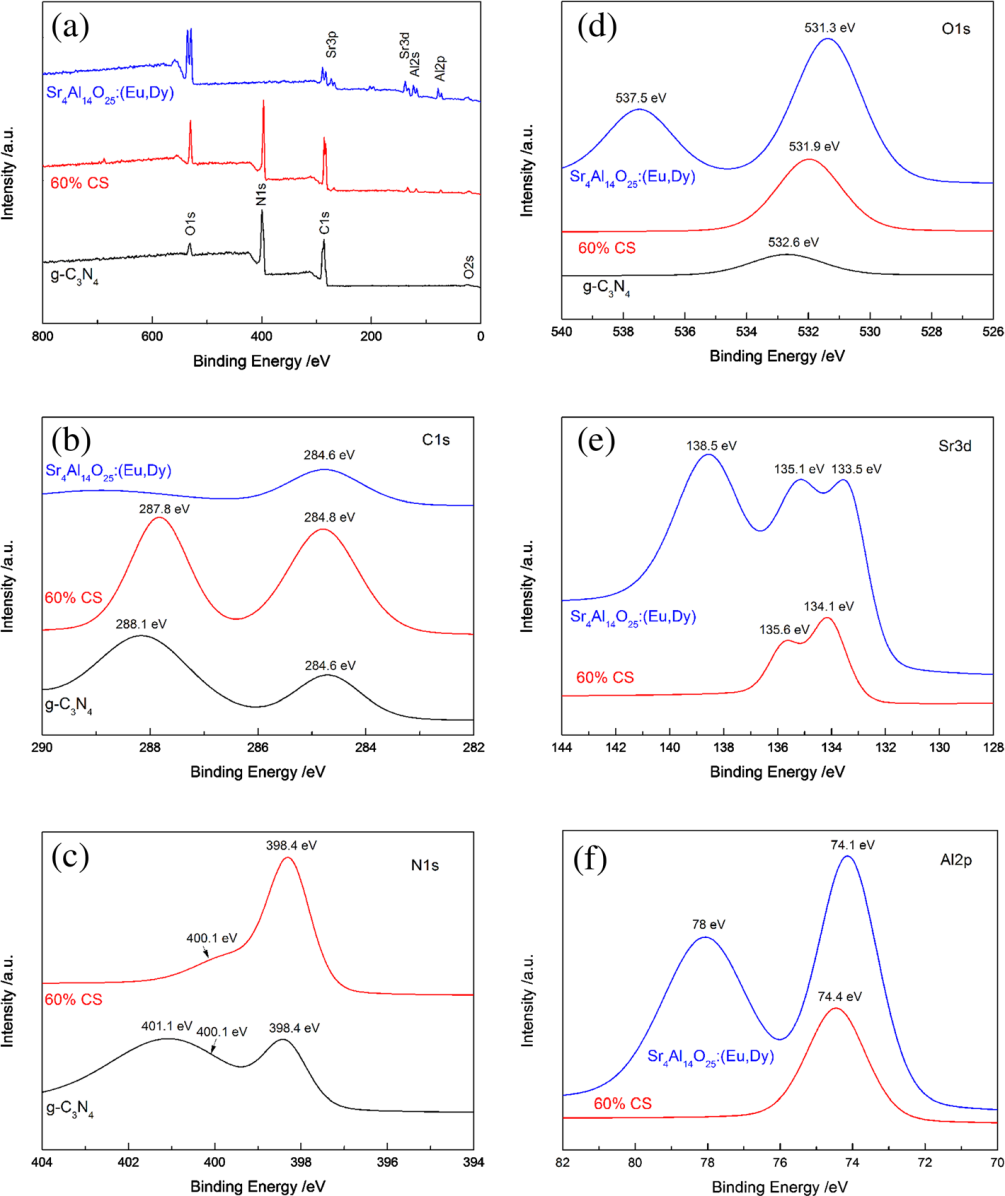
**a** XPS survey spectra of g-C_3_N_4_, Sr_4_Al_14_O_25_:(Eu,Dy), and 60 % CS composite; high resolution XPS spectra of the **b** C1s, **c** N1s, **d** O1s, **e** Sr3d, and **f** Al2p peak of g-C_3_N_4_, Sr_4_Al_14_O_25_:(Eu,Dy), and 60 % CS composite

The surface chemical compositions of g-C_3_N_4_, Sr_4_Al_14_O_25_:(Eu,Dy), and 60 % CS composite were analyzed by XPS. In Fig. 4a, the survey scanned XPS spectra provide C1s, N1s, and O1s peaks for g-C_3_N_4_ and 60 % CS, as well as Sr 3d, Al 2p, and O 1 s peaks for Sr_4_Al_14_O_25_:(Eu,Dy) and 60 % CS, which agree with chemical composition of the composites. As shown in the high-resolution XPS spectra of C 1 s (Fig. 4b), only one peak at 284.6 eV, which belongs to external carbon contamination is observed in the Sr_4_Al_14_O_25_:(Eu,Dy) sample. In case of g-C_3_N_4_, two carbon peaks at 284.6 and 288.1 eV are found. The first peak is assigned to external carbon atoms deposition on its surface [26, 28]. The second peak is related to a C–N–C bond in the g-C_3_N_4_ lattice [33].

The 60 % CS hybrid material also displays two C1s peaks at 284.8 and 287.8 eV, indicating a little difference in the binding energies compared with pure g-C_3_N_4_. It suggests that Sr_4_Al_14_O_25_:(Eu,Dy) hybridization with g-C_3_N_4_ resulted in an inner shift of the C1s orbit. The N1s peaks of g-C_3_N_4_ and 60 % CS are observed at 401.1, 400.1, and 398.4 eV in Fig. 4c. The main signal shows the occurrence of C–N–C bond (398.4 eV) and tertiary nitrogen N-(C)_3_ groups (400.1 eV) in g-C_3_N_4_ and 60 % CS. It also reveals an additional signal at 401.1 eV, indicative of the amino functions carrying hydrogen (C–N–H). In addition, the N1s peak maintained the same binding energy which suggests that a suitable combination of g-C_3_N_4_ and Sr_4_Al_14_O_25_:(Eu,Dy) inducing the N1s orbit offset can be ruled out. The O1s peak at 537.5 and 531.3 eV shown in Fig. 4d can be assigned to the O element in the Sr_4_Al_14_O_25_:(Eu,Dy).

At the same time, the O1s peak at 532.6 eV of g-C_3_N_4_ is derived from the hydroxyl group. After the combination with Sr_4_Al_14_O_25_:(Eu,Dy), the peak blue shifts to 531.9 eV, indicating there is the chemical bond between them. To further observe the chemical interaction, the Sr 3d XPS spectra are shown in Fig. 4e. It can be seen that the Sr 3d peak at 138.5 eV can be only found in pure Sr_4_Al_14_O_25_:(Eu,Dy), as well as the Sr 3d peaks at 133.5 and 135.1 eV for pristine Sr_4_Al_14_O_25_:(Eu,Dy) and 60 % CS hybrid shift to 134.1 and 135.6 eV, respectively, firmly confirming the interaction between the two components.

The red shift of the Sr 3d value indicates that the interaction can increase the effective negative charge of the Sr species. It is also supported by the result that the g-C_3_N_4_ possesses the surface hydroxyl groups. A similar phenomenon is also found in the XPS spectra of the Al 2p (Fig. 4f). The binding energies of the Al 2p (74.1 and 78 eV) of pure Sr_4_Al_14_O_25_:(Eu,Dy) are higher than that (74.4 eV) of 60 % CS hybrid. Such results can be similarly attributed to the interaction of g-C_3_N_4_ with Sr_4_Al_14_O_25_:(Eu,Dy), resulting in an inner shift of the Al 2p orbit. The analyses distinctly indicate the presence of chemical bonds between g-C_3_N_4_ and Sr_4_Al_14_O_25_:(Eu, Dy), rather than a simple physical mixing.

**Fig. 5:**
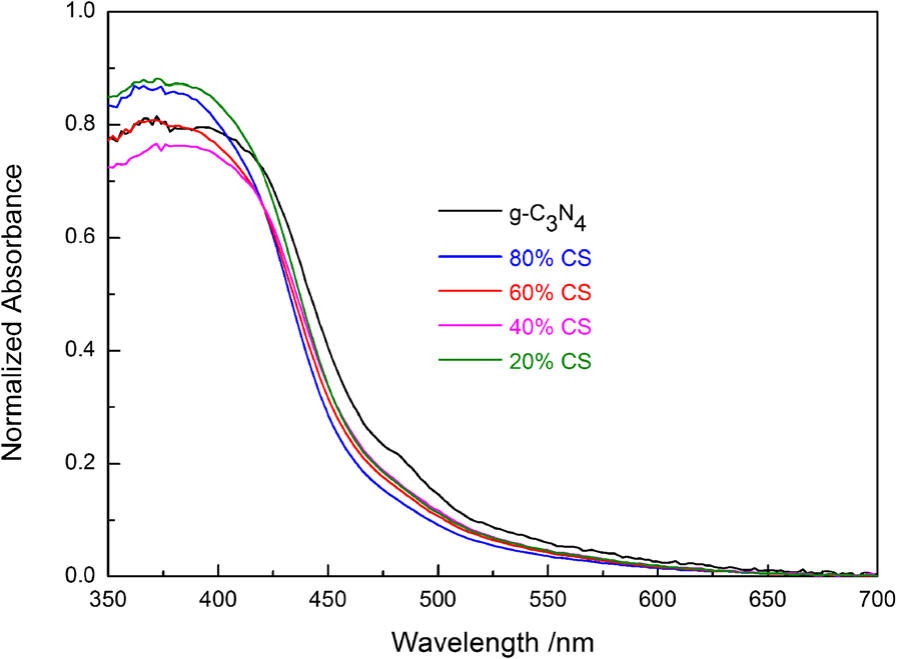
UV-vis diffuse reflectance spectra of the g-C_3_N_4_ and Sr_4_Al_14_O_25_:(Eu,Dy)/g-C_3_N_4_ composites

The UV-vis diffuse reflectance spectra of the samples are shown in Fig. 5. It is clear that the absorption intensity of pure g-C_3_N_4_ rises greatly at around 450 nm, in good accordance with the band gap of g-C_3_N_4_ (2.7 eV). For CS composites, compared to pure g-C_3_N_4_, the light absorption threshold of the hybrids is not changed; however, the absorption strength weakens due to the decrease of g-C_3_N_4_ content.

**Fig. 6:**
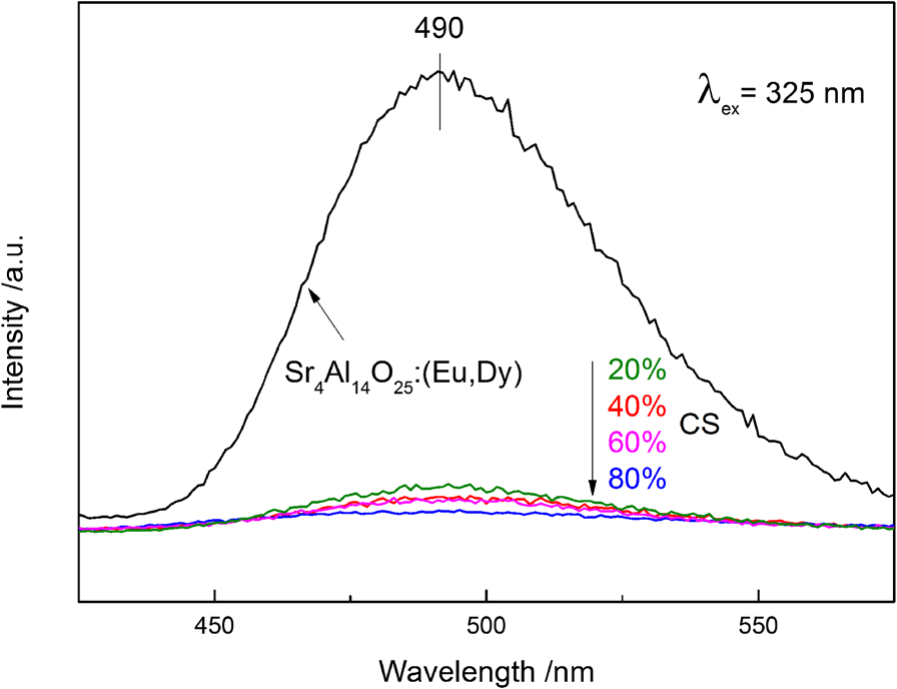
Emission spectra of Sr_4_Al_14_O_25_:(Eu,Dy) and various Sr_4_Al_14_O_25_:(Eu,Dy)/g-C_3_N_4_ composites

The emission spectra of Sr_4_Al_14_O_25_:(Eu,Dy) and various Sr_4_Al_14_O_25_:(Eu,Dy)/g-C_3_N_4_ composites under 325 nm excitation are displayed in Fig. 6. The emission spectra are identical in shape, although differ in intensities. The broadband emission centered at 490 nm can be assigned to the typical 4f^6^5d^1^-4f^7^ transition of Eu^2+^. Since the crystal field can greatly affect the 4f^6^5d^1^ electron states of Eu^2+^, it suggests that the crystal field is not changed much with the compositional variation. It is worthwhile to note that the phosphorescent property of Sr_4_Al_14_O_25_:(Eu,Dy) was retained even after deposition of a large amount of g-C_3_N_4_ on the surface although the PL intensity decreases with an increase in the g-C_3_N_4_ content. The XRD patterns (Fig. 1) show the similar result, in which the intensity of XRD peaks attributed to Sr_4_Al_14_O_25_:(Eu,Dy) decreased with an increase in the g-C_3_N_4_ content. The phosphorescence with the wavelength of 490 nm generated from Sr_4_Al_14_O_25_:(Eu,Dy) can be used for photocatalytic reaction on g-C_3_N_4_, since the g-C_3_N_4_ can absorb the visible light up to 550 nm of wavelength.

**Fig. 7:**
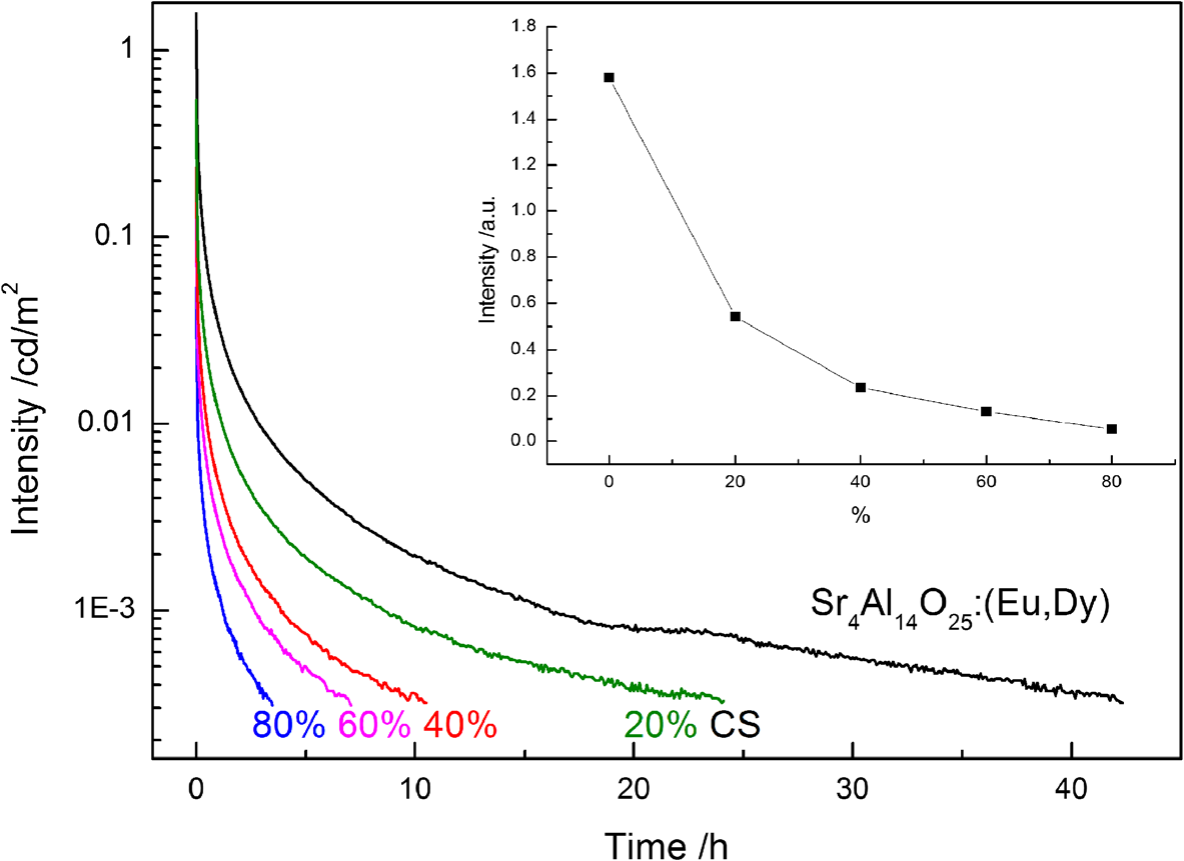
Decay curves of fluorescence intensities of Sr_4_Al_14_O_25_:(Eu,Dy) and Sr_4_Al_14_O_25_:(Eu,Dy)/g-C_3_N_4_ composites. *Inset* shows the effect of the g-C_3_N_4_ content on the initial fluorescence intensity

Figure 7 presents decay curves of fluorescence intensities of Sr_4_Al_14_O_25_:(Eu,Dy) and Sr_4_Al_14_O_25_:(Eu,Dy)/g-C_3_N_4_ composites. Each of them consists of a fast decay and a consequent slow decay with a long decay tail [34]. The initial fluorescence intensity and duration vary largely for different composition. With the increase of g-C_3_N_4_ content, both the intensity and the duration of afterglow decrease gradually, but its afterglow property is still excellent, i.e., the afterglow of even 80 % CS can last for 3.5 h. This clearly indicates the utilization potential of the long afterglow as a light source for photocatalytic reaction in the dark.

**Fig. 8:**
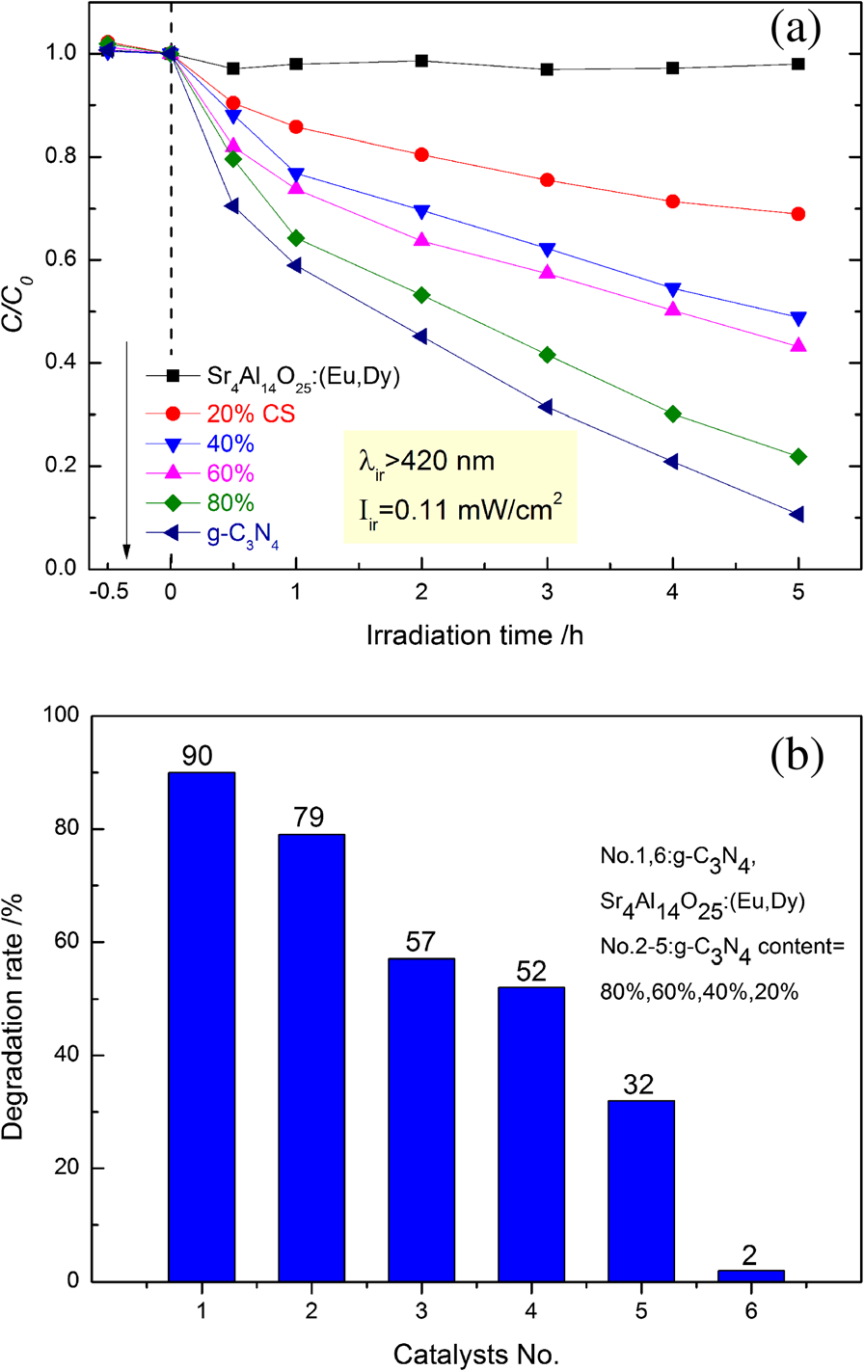
**a** Decomposition of the MB dye with time in the presence of Sr_4_Al_14_O_25_:(Eu,Dy), g-C_3_N_4,_ and various Sr_4_Al_14_O_25_:(Eu,Dy)/g-C_3_N_4_ composites under visible-light irradiation; **b** Degradation rate of MB after the 5 h irradiation of visible-light

The visible light (*λ* > 420 nm)-induced photocatalytic activities of g-C_3_N_4_, Sr_4_Al_14_O_25_:(Eu,Dy) and Sr_4_Al_14_O_25_:(Eu,Dy)/g-C_3_N_4_ composites were evaluated by comparing the degradation rates of the MB dye (Fig. 8). From Fig. 8a, the MB dye is gradually decomposed with time in the presence of g-C_3_N_4_ and various Sr_4_Al_14_O_25_:(Eu,Dy)/g-C_3_N_4_ composites. In contrast, the degradation of MB in the presence of Sr_4_Al_14_O_25_:(Eu,Dy) is negligibly small, suggesting that MB dye is stable, and the photocatalytic activity of Sr_4_Al_14_O_25_:(Eu,Dy) is small. After the 5 h of visible-light irradiation, 90 % of MB removal is achieved over pure g-C_3_N_4_. All composites exhibit the photocatalytic activity markedly lower than pure g-C_3_N_4_ under visible-light irradiation, i.e., with an increase in g-C_3_N_4_ content from 20 to 80 wt%, the removal rates of MB increase from 32 to 79 % under visible-light irradiation for 5 h. For the composites, the photocatalytic activity looks like simply related to g-C_3_N_4_ content.

**Fig. 9:**
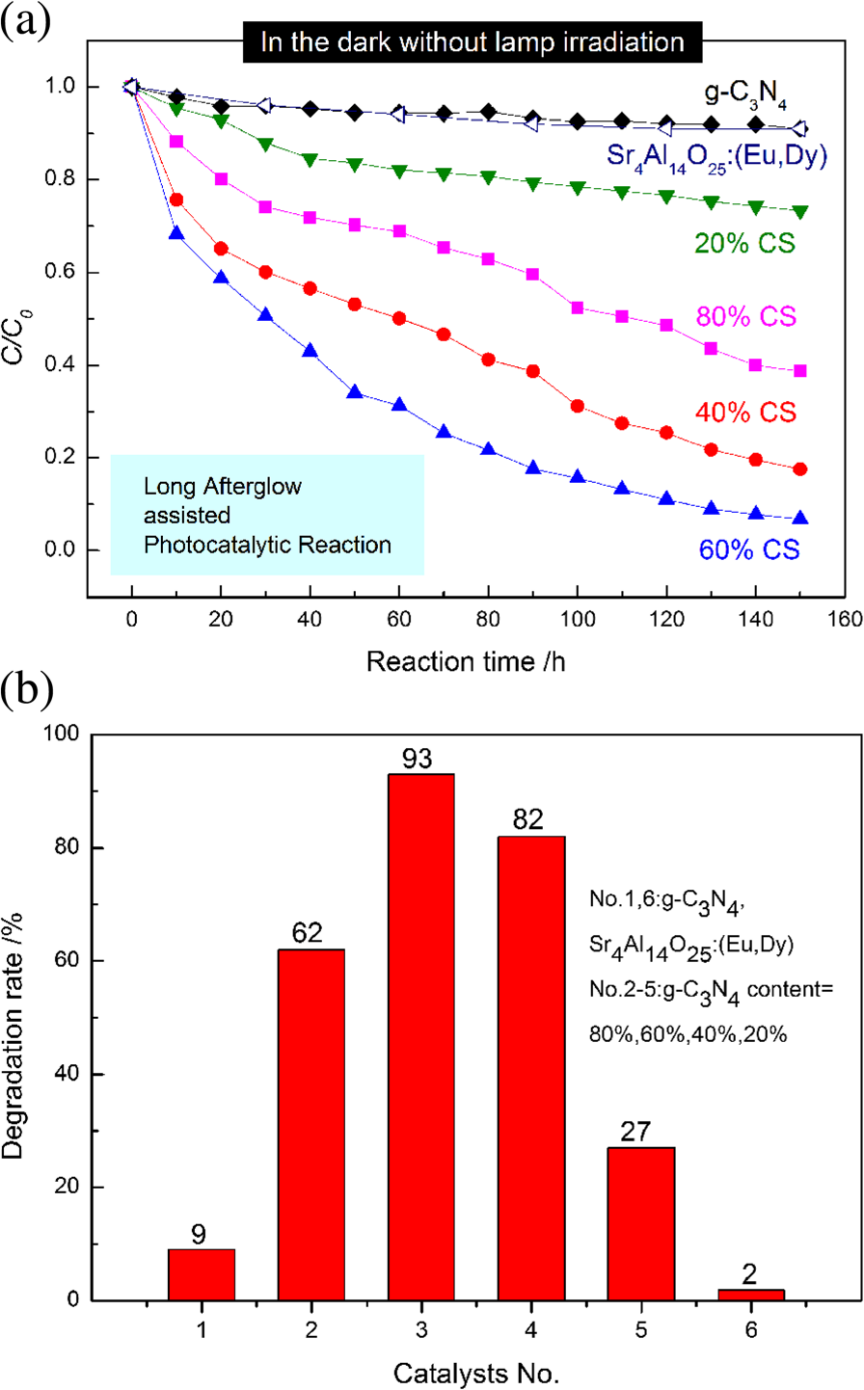
**a** Decomposition of MB by the repeated reactions in the dark by adding various samples irradiated by an 8-W black lamp for 30 min. Each photocatalytic reaction time was 10 h and 10 times of reactions were conducted. **b** Degradation rate of MB after 150 h irradiation (10 times of repeated reactions)

In contrast, the different phenomenon was observed when the photocatalytic reaction was conducted in the dark with the assistance of the long afterglow. The photocatalytic behaviors of Sr_4_Al_14_O_25_:(Eu,Dy)/g-C_3_N_4_ composites for the degradation of the MB dyes in the dark were explored by the repeated irradiation method (Fig. 9). For comparison, the performances of the pure g-C_3_N_4_ particles were also investigated. It is worth mentioning that both pure and composite samples showed so weak physical adsorptions of the MB dye; therefore, the removal of the MB dye by the physical adsorption is negligibly small. As expected, all the Sr_4_Al_14_O_25_:(Eu,Dy)/g-C_3_N_4_ composites exhibit good photocatalytic performance for the MB decomposition, while the decompositions of MB in the presence of pure g-C_3_N_4_ (only 9 % removal) and Sr_4_Al_14_O_25_:(Eu,Dy) (only 2 % removal) are quite small. These results suggest that the long afterglow generated by Sr_4_Al_14_O_25_:(Eu,Dy) acts as a light source to excite g-C_3_N_4_ and proceed the photocatalytic decomposition of the MB dye. Therefore, the combination of the visible-light responsive g-C_3_N_4_ and long afterglow phosphor such as Sr_4_Al_14_O_25_:(Eu,Dy) is essential to proceed the photocatalytic reaction in the dark. Thereby, the degradation of organic dyes in the dark may proceed efficiently by using a composite with the appropriate mass ratio of long afterglow phosphor and g-C_3_N_4_ photocatalyst. Under the present reaction conditions, the 60 % CS composite shows the highest photocatalytic activity, i.e., it can degrade 93 % of the MB dye in the dark for 150 h with the phosphorescence assistance. The composites with a larger content of g-C_3_N_4_, such as 80 % CS and pure g-C_3_N_4_, show lower photocatalytic activity in the dark without lamp irradiation. It can be owing to the insufficiency of light. On the other hand, in composites with zero or low intake of g-C_3_N_4_, large amount of Sr_4_Al_14_O_25_:(Eu,Nd) provided as reaction light cannot replace the important role of photocatalyst in the photocatalytic reaction progress. It results in the low or no photocatalytic activity.

## Conclusions

In summary, a facile and efficient process for the MB dye degradation in the dark has been achieved over the Sr_4_Al_14_O_25_:(Eu,Dy)-assisted g-C_3_N_4_ composite photocatalysts. Compared with pure g-C_3_N_4_, the hybrids with long afterglow phosphor can dramatically realize the photocatalytic aqueous MB oxidation in the dark. The enhanced photocatalytic efficiency of composites is attributed to the long afterglow assistance from Sr_4_Al_14_O_25_:(Eu,Dy) and the visible-light absorptivity of g-C_3_N_4_. Moreover, the studies of the photocatalytic performance of the hybrid photocatalysts revealed that the Sr_4_Al_14_O_25_:(Eu,Dy)/g-C_3_N_4_ composite consisting of 60 wt% g-C_3_N_4_ exhibited the highest catalytic activity in the dark. Considering the high price and low photochemical stability of Ag_3_PO_4_, the metal free g-C_3_N_4_ photocatalyst displays the desirable potential in the field of long afterglow-assisted photocatalysis in the dark. This work opens a new avenue for the development of g-C_3_N_4_ photocatalyst for the environmental cleanup.

## References

[1] Zhang Y, Su P, Huang J, Wang Q, Zhao B (2015). A magnetic nanomaterial modified with poly-lysine for efficient removal of anionic dyes from water. Chem Eng J.

[2] Mohan S, Rao N, Prasad K, Karthikeyan J (2002). Treatment of simulated Reactive Yellow 22 (Azo) dye effluents using Spirogyra species. Waste Manag.

[3] Ali N, Hameed A, Ahmed S (2009). Physicochemical characterization and bioremediation perspective of textile effluent, dyes and metals by indigenous bacteria. J Hazard Mater.

[4] Alver E, Metin A (2012). Anionic dye removal from aqueous solutions using modified zeolite: adsorption kinetics and isotherm studies. Chem Eng J.

[5] Hariharasuthan R, Rao A, Bhaskaran A (2013). Adsorption studies on reactive blue 4 by varying the concentration of Mgo In Sorel’s cement. Adsorption.

[6] Khataee AR, Safarpour M, Naseri A, Zarei M (2012). Photoelectro-Fenton/nanophotocatalysis decolorization of three textile dyes mixture: response surface modeling and multivariate calibration procedure for simultaneous determination. J Electroanal Chem.

[7] Zhang Y, Han C, Zhang G, Dionysiou D, Nadagouda M (2015). PEG-assisted synthesis of crystal TiO_2_ nanowires with high specific surface area for enhanced photocatalytic degradation of atrazine. Chem Eng J.

[8] Xu S, Fu L, Pham T, Yu A, Han F, Chen L (2015). Preparation of ZnO flower/reduced graphene oxide composite with enhanced photocatalytic performance under sunlight. Ceram Int.

[9] Sluaeman U, Yin S, Sato T (2011). Solvothermal synthesis and photocatalytic properties of chromium-doped SrTiO_3_ nanoparticles. Appl Catal B Environ.

[10] Ji Y, Cao J, Jiang L, Zhang Y, Yi Z (2014). G-C_3_N_4_/BiVO_4_ composites with enhanced and stable visible light photocatalytic activity. J Alloys Compd.

[11] Chen X, Liu L, Yu P, Mao S (2011). Increasing solar absorption for photocatalysis with black hydrogenated titanium dioxide nanocrystals. Science.

[12] Yi Z, Ye J, Kikugawa N, Kako T, Ouyang S, Williams H, Yang H, Cao J, Luo W, Li Z, Liu Y, Withers R (2010). An orthophosphate semiconductor with photooxidation properties under visible-light irradiation. Nat Mater.

[13] Guan X, Guo L (2014). Cocatalytic effect of SrTiO_3_ on Ag_3_PO_4_ toward enhanced photocatalytic water oxidation. ACS Catal.

[14] Reunchan P, Umezawa N (2015). Sulfur and silicon doping in Ag_3_PO_4_. J Phys Chem C.

[15] Bi Y, Ouyang S, Umezawa N, Cao J, Ye J (2011). Facet effect of single-crystalline Ag_3_PO_4_ sub-microcrystals on photocatalytic properties. J Am Chem Soc.

[16] Li H, Yin S, Wang Y, Sekino T, Lee S, Sato T (2013). Green phosphorescence-assisted degradation of rhodamine B dyes by Ag_3_PO_4_. J Mater Chem A.

[17] Bi Y, Quyang S, Cao J, Ye J (2011). Facile synthesis of rhombic dodecahedral AgX/Ag_3_PO_4_ (X = Cl, Br, I) heterocrystals with enhanced photocatalytic properties and stabilities. Phys Chem Chem Phys.

[18] Ma X, Li H, Wang Y, Li H, Liu B, Yin S, Sato T (2014). Substantial change in phenomenon of “self-corrosion” on Ag_3_PO_4_/TiO_2_ compound photocatalyst. Appl Catal B Environ.

[19] Sun S, Wang W, Zhang L, Shang M (2009). Visible light-induced photocatalytic oxidation of phenol and aqueous ammonia in flowerlike Bi_2_Fe_4_O_9_ suspensions. J Phys Chem C.

[20] Wang L, Wang W, Shang M, Sun S, Yin W, Ren J, Zhou J (2010). Visible light responsive bismuth niobate photocatalyst: enhanced contaminant degradation and hydrogen generation. J Mater Chem.

[21] Sun S, Wang W, Zhang L (2012). Facile preparation of three-dimensionally ordered macroporous Bi_2_WO_6_ with high photocatalytic activity. J Mater Chem.

[22] Wang H, Su Y, Zhao H, Yu H, Chen S, Zhang Y, Quan X (2014). Photocatalytic oxidation of aqueous ammonia using atomic single layer graphitic-C_3_N_4_. Environ Sci Technol.

[23] Wang X, Maeda K, Thomas A, Takanabe K, Xin G, Carlsson J, Domen K, Antonietti M (2009). A metal-free polymeric photocatalyst for hydrogen production from water under visible light. Nat Mater.

[24] Su F, Mathew S, Lipner G, Fu X, Antonietti M, Blechert S, Wang X (2010). mpg-C_3_N_4_-catalyzed selective oxidation of alcohols using O_2_ and visible light. J Am Chem Soc.

[25] Xu J, Wang Y, Zhu Y (2013). Nanoporous graphitic carbon nitride with enhanced photocatalytic performance. Langmuir.

[26] Li F, Zhao Y, Wang Q, Wang X, Hao Y, Liu R, Zhao D (2015). Enhanced visible-light photocatalytic activity of active Al_2_O_3_/g-C_3_N_4_ heterojunctions synthesized via surface hydroxyl modification. J Hazard Mater.

[27] Yan S, Lv S, Li Z, Zou Z (2010). Organic-inorganic composite photocatalyst of g-C_3_N_4_ and TaON with improved visible light photocatalytic activities. Dalton Trans.

[28] Wang S, Li D, Sun C, Yang S, Guan Y, He H (2014). Synthesis and characterization of g-C_3_N_4_/Ag_3_VO_4_ composites with significantly enhanced visible-light photocatalytic activity for triphenylethane dye degradation. Appl Catal B Environ.

[29] Kondo K, Murakamia N, Ye C, Tsubota T, Ohno T (2013). Development of highly efficient sulfur-doped TiO_2_ photocatalysts hybridized with graphitic carbon nitride. Appl Catal B Environ.

[30] He Y, Zhang L, Fan M, Wang X, Walbridge M, Nong Q, Wu Y, Zhao L (2015). Z-scheme SnO_2-x_/g-C_3_N_4_ composite as an efficient photocatalyst for dye degradation and photocatalytic CO_2_ reduction. Sol Energ Mat Sol C.

[31] Emen F, Kafadar V, Kulcu N, Yazici A (2013). Thermoluminescence studies and detailed kinetic analysis of Sr_4_Al_14_O_25_:Eu^2+^, Dy^3+^ phosphor. J Lumin.

[32] Wang Y, Shi R, Lin J, Zhu Y (2011). Enhancement of photocurrent and photocatalytic activity of ZnO hybridized with graphite-like C_3_N_4_. Energy Environ Sci.

[33] Thomas A, Fischer A, Goettmann F, Antonietti M, Mueller J, Schloegl R, Carlsson J (2008). Graphitic carbon nitride materials: variation of structure and morphology and their use as metal-free catalysts. J Mater Chem.

[34] Li Y, Wang Y, Xu X, Gong Y (2009). Effects of non-stoichiometry on crystallinity, photoluminescence and afterglow properties of Sr_2_MgSi_2_O_7_:Eu^2+^, Dy^3+^ phosphors. J Lumin.

